# Upregulation of Mrps18a in breast cancer identified by selecting phage antibody libraries on breast tissue sections

**DOI:** 10.1186/s12885-016-2987-5

**Published:** 2017-01-05

**Authors:** Karen Marie Juul Sørensen, Theresa Meldgaard, Connie Jenning Melchjorsen, Agla J. Fridriksdottir, Henrik Pedersen, Ole William Petersen, Peter Kristensen

**Affiliations:** 1 Department of Engineering, Aarhus University, Gustav Wieds Vej 10, 8000 Aarhus C Aarhus, Denmark; 2 Department of Cellular and Molecular Medicine, Centre for Biological Disease Analysis and Danish Stem Cell Centre, University of Copenhagen, Copenhagen, Denmark

**Keywords:** Phage display, Domain antibodies, Breast cancer, Shadow stick selection, Mitochondrial ribosomal protein s18a

## Abstract

**Background:**

One of the hallmarks of cancer is an altered energy metabolism, and here, mitochondria play a central role. Previous studies have indicated that some mitochondrial ribosomal proteins change their expression patterns upon transformation.

**Method:**

In this study, we have used the selection of recombinant antibody libraries displayed on the surface of filamentous bacteriophage as a proteomics discovery tool for the identification of breast cancer biomarkers. A small subpopulation of breast cells expressing both cytokeratin 19 and cytokeratin 14 was targeted using a novel selection procedure.

**Results:**

We identified the mitochondrial ribosomal protein s18a (Mrps18a) as a protein which is upregulated in breast cancer. However, Mrps18a was not homogeneously upregulated in all cancer cells, suggesting the existence of sub-populations within the tumor. The upregulation was not confined to cytokeratin 19 and cytokeratin 14 double positive cells.

**Conclusion:**

This study illustrates how phage display can be applied towards the discovery of proteins which exhibit changes in their expression patterns. We identified the mitochondrial protein Mrps18a as being upregulated in human breast cancer cells compared to normal breast cells.

**Electronic supplementary material:**

The online version of this article (doi:10.1186/s12885-016-2987-5) contains supplementary material, which is available to authorized users.

## Background

Cancer is a prevalent cause of death, and is estimated to have been responsible for 8.2 million deaths globally in 2012. Accounting for approximately 521 000 deaths every year, breast cancer is one of the most common causes of cancer-related death [1].

Changes in cell metabolism are central to cancer development [2]. Mitochondria play a central role in regulating parameters of the metabolism, such as energy production, production of biosynthetic precursors, redox status, reactive oxygen species generation, cytosolic calcium levels, and the initiation of apoptosis. In both normal cells and cancer cells, changes in these parameters may prompt a shift in the cell state, e.g., from quiescent and differentiated to actively proliferating. Increases and decreases in mitochondrial activity can be mediated by mutations in genes encoding mitochondrial proteins. In the context of breast cancer, several studies on mitochondrial alterations are available and have been reviewed in [3]. Only a small fraction of mitochondrial proteins are encoded by the mitochondrial DNA. Nuclear DNA encodes all other mitochondrial proteins, including mitochondrial ribosomal proteins (Mrps). This emphasizes the integrated nature of the cross-talk between the mitochondria and the nucleus.

Early proteomic analysis of the mammalian mitochondrial ribosome reported three variants of Mrps18 [4, 5]. Although these early studies revealed that members of the s18 protein family are localized on the surface of the small subunit (S28) of the mitochondrial ribosome, the function of these proteins is largely unknown. Recent data reporting the complete structure of the large mitochondrial ribosomal subunit has revealed that Mrps18a is not located on the small subunit as previously indicated, but on the large ribosomal subunit [6], which demonstrates the lack of knowledge concerning the importance and function of Mrps18a. The three variants, designated Mprs18a, Mrps18b, and Mrps18c, are the products of three separate genes. The genes are only 25–30% identical, which is comparable to their homology with their bacterial counterparts [4]. It has been suggested that there may be different sub-populations of cells within tissues where the expression levels of the different Mrps18 variants vary [7]. In 2009, it was shown that overexpression of human Mrps18b in primary rat embryonic fibroblasts leads to immortalization. A cell line derived from these cells, called 18IM, adopted several cancer-like characteristics, such as loss of contact inhibition and anchorage-independent growth. Furthermore, 18IM cells acquired expression of different stem cell markers [8]. Microarray and Q-PCR analysis of 18IM and three other equivalent cell lines showed up-regulation of a range of genes involved in mitochondrial pathways such as energy production, which are characteristic of rapidly proliferating cells [9]. Recently, a study of overexpression of Mrps18b in human breast and renal cancer cell lines has shown that Mrps18b results in the appearance of multinucleated cells [10]. These observations indicate that Mrps18 family proteins, or at least Mrps18b, may be involved in neoplastic transformation.

It is widely believed that intertumor heterogeneity reflects the “cell of origin” as well as the mutational profile of a given cancer cell [11, 12]. Much effort has been put into unravelling the normal cellular hierarchy of the human breast, as it is an important step toward understanding the “cells of origin” and molecular events that drive breast cancer. Several populations of cells within the human breast have been proposed as candidates for the “cells of origin” of cancer, including stem cells and progenitors, or transit-amplifying cells. One such candidate population was identified in a study performed by Villadsen et al., and supported by Honeth et al. [13], where a putative stem cell zone was identified in the ducts of the normal human breast containing multipotent cells positive for both the luminal epithelial lineage marker, cytokeratin 19 (K19), and the myoepithelial lineage marker, cytokeratin 14 (K14) [14].

In this study, we aimed to identify proteins which are differentially regulated in breast cancer and may therefore have a potential use in the diagnosis and/or treatment of breast cancer. Phage display-mediated selections of human domain antibodies were performed against ductal regions containing K19^+^/K14^+^ cells in normal breast tissue cryostat sections using the shadow stick selection technique [15, 16] adopted from [17–19]. Here, a recombinant antibody library, displayed in genetic fusion with the filamentous bacteriophage protein 3 [20], is allowed to bind to antigens presented on the tissue sections. Compared to selection on intact cells obtained from circulation or cell culture, the tissue sections allow all cellular compartments to be targeted, as the sections normally have a thickness of 4–8 μM. Following removal of non-binding phage antibodies by washing, the target cells in the tissue are covered by a shadow stick and the tissue section is illuminated by UV-C light. The UV-C light causes crosslinking in the phage genome, resulting in phage particles incapable of replication after infection. Phage particles covered by the shadow stick are protected from the UV-C light and remain able to replicate after infection. In this way, phage display selection becomes a discovery tool in the search for proteins which are differentially regulated between distinct cell populations, which may represent novel biomarkers.

A potentially interesting antigen, Mrps18a, emerged as the cognate antigen of one of the selected phage antibodies. Although the functions of this particular protein are currently unexplored both in the context of the normal cell state as well as in cancer cells, we found that the expression of Mrps18a is upregulated in breast cancer cells compared to normal cells. Mrps18a cannot be considered a unique biomarker for K19 and K14 positive cells, as it is found in both normal and cancer cells. However, its increased expression in cancer cells is explained by the increased energy metabolism of cancer cells, and it may pave the way for new diagnostic and therapeutic routes to be explored.

## Methods

### Tissue sections

Biopsies of healthy human breast tissue and breast cancer tissue were obtained from five patients (P636, P659, P722, P727, and P819) undergoing reduction mammoplasty and three cancer patients (P757, P942, and P949), respectively. The use of human material has been reviewed by the Regional Scientific Ethical Committees (Region Hovedstaden) and approved with reference to H-2-2011-052 and H-2-2010-051. Informed consent was obtained from each patient regarding the collection and use of tissue. Cryostat sections (8 μm) were prepared as described by [21]. All tissue sections were fixed in methanol for 5 min at −20 °C.

### Target area identification

In preparation for selection, tissue sections were stained by immunohistochemistry (IHC) with antibodies against K19 and K14 and detected with Alexa Flour A568 and A488 conjugated isotype specific goat anti-mouse antibodies, respectively. For details on the antibodies, see Tables 1 and 2.

**Table 1:** Primary antibodies used for IHC

Description	Isotype	Dilution	Source
Mouse monoclonal Anti-K14	IgG3	1:50	Leica Biosystems, NCL-L-LL002
Mouse monoclonal Anti-K19	IgG2a	1:100	Abcam, ab7754
dAb	-	~250 μg/mL	-
dAb-rFc	-	40–100 μg/mL	-

**Table 2:** Secondary antibodies used for IHC

Description	Flourophore	Isotype specificity	Dilution	Source	Detection of
Goat anti-mouse	A488	IgG_3_	1:500	Invitrogen, A21151	CK14
Goat anti-mouse	A350	IgG_2a_	1:250	Invitrogen, A21130	CK19
Goat anti-mouse	A488	IgG_2a_	1:500	Invitrogen, A21131	CK19
Mouse anti-human c-Myc	Cy3	IgG_1_	1:250	Sigma-Aldrich, C6594	dAb
Goat anti-rabbit	A488	IgG (H + L)	1:400	Invitrogen, A11008	dAb-rFc

*A* Alexa Fluor^®^ fluorescent dye

One tissue section from each patient was mounted with Fluoromount (Sigma-Aldrich), and areas containing rare K19^+^/14^+^ cells were identified. Only patient samples containing 1–2 areas with 2–10 luminal positioned K19^+^/14^+^ cells residing in ductal regions were used for selections.

### Selection

The tissue section was blocked for 1 h in 4% Marvel dried skim milk powder (MPBS). The tissue section was then incubated with the Predator single domain library [22] in a slide container containing 20 mL 2% MPBS for 1½ h on a turntable under gentle agitation and for 1½ h without agitation. Approximately 10^12^ phage particles of the Predator library were applied [22]. The tissue section was washed twice for 10 min in PBS and twice for 10 min in PBS with 10% glycerol (PBSG) under gentle agitation. The tissue section was dried except the target area, which was kept moist with PBSG. Using brightfield microscopy, a custom made shadow stick (~80 μm) was positioned above the target area. Shadow sticks were prepared as previously described [23]. The tissue section was exposed to UV-C light (254 nm) for 10 min using a UV-C source (model UVSL-14P from UVP, Upland) positioned approximately 5 cm above the tissue section. Phage particles were eluted with 15 μL trypsin (1 mg/mL) for 10 min. Trypsin was aspirated and transferred to 50 μL ice cold foetal bovine serum. Subsequently, the target area was washed 15 times with 50 μL PBSG. This liquid was transferred to the eluate before storage overnight at −20 °C. The trypsinated phages were propagated in *E. coli* and single colonies were cultured in a 96-well plate as described by [23].

### Phage antibody ELISA

Phage antibodies were produced by superinfection with the KM13 helper phage [24]. This was performed in 96-well format for screening and in 50 mL cultures for ELISA with dilution arrays of monoclonal phage antibodies respectively followed by precipitation with 20% poly ethylene glycol (PEG) 6000, 2.5 M NaCl. Phage titers from 50 mL cultures were estimated by calculation using absorbance measured at 269 and 320 nm as described by [25].

Costar 96 well culture plates (Corning) with luminal breast cells enriched for K19^+^/K14^+^ cells were prepared by FACS and short term culturing. Luminal cells were initially sorted from normal breast tissue (source as describes for tissue sections) with EpCAM, then cultured in FAD2 medium consisting of Ham’s F12 : DMEM mixed in a 1:3 ratio (Tan DW, 2013), and 5% FCS (Liu X, 2012). The cells were cultured on a feeder layer of mouse fibroblasts to minimize the arisal of basal K14^+^ cells. Sorting of EpCAM^+^/CD271^+^ cells from these cultures resulted in a population enriched for K19^+^/K14^+^ cells. These were cultured in FAD2 medium in collagen coated flasks. K19^+^/K14^+^ and K19^+^ cells were represented approximately 1:1. The cells were seeded in 96-wells, approximately 10–20 000 cells/well, and cultured 3–5 days before fixation for 10 min with 3.7% formalin, followed by 10 min with 0.1% triton X-100. The plates were dried overnight at 37 °C.

For screening, 50 μl 4% MPBS and 50 μL of the individual PEG precipitated phage antibodies were transfered to a cell coated 96-well plate. For ELISA with dilution arrays of monoclonal phage antibodies, a six pointed 10-fold dilution series was prepared. From this, 100 μL was transferred to the 96-well plate resulting in concentrations ranging from 10^11^ phages/well to 10^7^ phages/well. As a positive control, the phage scFv antibody 52 was used [26]. This phage antibody has been reported to have affinity towards at least 14 different cell lines, among these breast cancer cell line MCF7 [26]. As a negative control, a phage scFv antibody specific to foetal Epsilon-haemoglobin, DAb1, was included [27]. For both screening and ELISA with dilution arrays, bound phage antibodies were detected with HRP conjugated M13 phage antibody (GE healthcare) 1:3000 and TMB Plus “Ready-to-use” substrate solution (Thermo, Fischer Scientific) according to the manufacturer’s protocol. The chromogenic reaction was stopped after 10–15 min by addition of 50 μL 1 M H_2_SO_4_. Absorbance (OD_450_) was read on a Bio-Rad Model 550 microplate reader (Bio-Rad) with subtraction of background (OD_655_).

### Expression of single domain antibody (dAb) in *E. coli*

For expression of soluble dAb, the sequence encoding BC5 was sub-cloned from phagemid into pET22b-MycHis, a modified version of pET22b (Merck, KGaA), using *NcoI* and *NotI* (FastDigest, Fermentas) according to the manufacturer’s protocol. This expression plasmid was transformed into BL21 Gold DE3 (Aglient Technologies). For expression, 200 mL exponentially growing cultures were set up in TB growth medium supplemented with 100 μg/mL ampicillin and 4% w/v glucose at 37 °C, 200 rpm. At an OD_600_ of approximately 0.6–0.8, protein expression was induced by exchange of growth medium to TB containing 100 μg/mL ampicillin and 1 μM IPTG and incubated 18–20 h at 18 °C, 200 rpm. Protein was precipitated using 30% w/v ammonium sulphate. Pelleted protein was resuspended in 20 mL Protein A binding buffer TBS (50 mM Tris, 150 mM NaCl) pH 8.0 added 0.1 mM DNase (Roche). The protein suspension was sterile filtered (0.45 μm) before affinity purification on HiTrap Protein A column (GE Healthcare) according to the manufacturer’s protocol, except eluted with 2.5% acetic acid. Purified antibody fragments were transferred to TBS, pH 7 by dialysis. A purity over 95% was confirmed by SDS-PAGE, and the protein concentration was assessed with a NanoDrop 1000 (Thermo, Fisher Scientific).

### Expression of dAb with rabbit Fc-domain (dAb-rFc) in *L. tarentolae* T7-TR

In order to express soluble dAb-rFc, the sequence encoding BC5 antibody was sub-cloned from phagemid into pMJ_LEXSY-rFc vector, and expressed and purified as described [28].

### Immunohistochemistry

For initial IHC, tissue sections were blocked in Ultravision Block (Thermo scientific) for 1 h. Incubation with primary antibody (Table 1) in a total volume of 100 μL Ultra Vision Block with 10% goat serum (Sigma Aldrich) was performed overnight at 4 °C in a humidity box. Incubation with fluorescent secondary antibodies (Table 2) were performed for 30 min in 100 μL Ultra Vision Block with 10% goat serum. Flouromount (Sigma-Aldrich) was used for mounting. A frozen tissue microarray with 5 μm sections (BioChain^®^, Cat. T6235086-1) was blocked and stained as described above. Fluorescence microscopy was performed with a Leica DMI 3000 B microscope with Cell B imaging software.

### Antigen identification–protein macroarray

Screening of a UniPEx1 protein macroarray (BioScience ImaGenes) was performed according to the manufacturer’s protocol with purified recombinant antibody, either dAb or dAb-rFc. Briefly, the activated membrane was blocked with 5% BSA for 1 h before incubation overnight with 250 μg dAb or 125 μg dAb-rFc. Detection was performed by incubation for 2 h with secondary antibody; Monoclonal Anti-c-Myc − Cy3™ (Sigma-Aldrich) 1:2000 or Goat anti-Rabbit-IgG-Cy5 (Abcam, ab6564) 1:8000. The membrane was scanned with a Typhoon TRIO variable mode imager (Amersham Biosciences). Antibody binding to the proteins on the array was evaluated using an in-house developed Matlab program. The immobilised proteins were situated on the membrane in defined duplicated patterns arranged in squares consisting of 3x3 spots with a central ink dot. By evaluating the intensities of different spots compared to the background, as well as their positions, the program created a list of possible antigen hits listed from highest to lowest intensity. Only hits detected with both dAb and dAb-rFc formats were considered. Furthermore, hits detected only once despite being spotted several times in duplex were omitted.

### Indirect ELISA with Mrps18a

MRPS18A cDNA was purchased (Source BioScience, IRAUp969D0453D) and the sequence verified. The insert was sub-cloned into pETM11 after PCR amplification using primers inserting recognition sequences for the restriction enzymes *NotI* and *NcoI*. This allowed for cytosolic expression in BL21 Gold DE3 and subsequent affinity purification under native conditions on Ni-NTA Superflow Beads (QIAGEN) according to manufacturer’s protocol.

A 96-well MaxiSorp plate (Thermo, Fisher Scientific) was coated with purified Mrps18a in concentrations ranging from 1 to 1500 ng in TBS, pH 7.5, per well and blocked with 2% BSA. The plate was incubated for 2.5 h under gentle agitation with 100 μg/mL or 200 μg/mL dAb. Bound antibodies were detected with HRP conjugated Anti-c-Myc (not commercial) 1:400. TMB “Ready-to-use” substrate was applied as described above.

### Far western blot analysis

Purified Mrps18a, was run in a standard SDS-PAGE under reducing conditions and then electro blotted onto a PVDF membrane using the traditional sandwich method. Denaturing and renaturing of protein in the membrane was facilitated by subsequent steps of incubation with AC buffers with decreasing concentrations of guanidine–HCl (6 M–0 M) as described [29]. Subsequently, the membrane was incubated with 10 μg BC5 (dAb-rFc), followed by HRP conjugated swine anti-rabbit (DAKO) 1:4000. Detection was carried out with an ECL detection kit (Amersham Biosciences).

## Results

### Selection using the shadow stick method

**Fig. 1:**
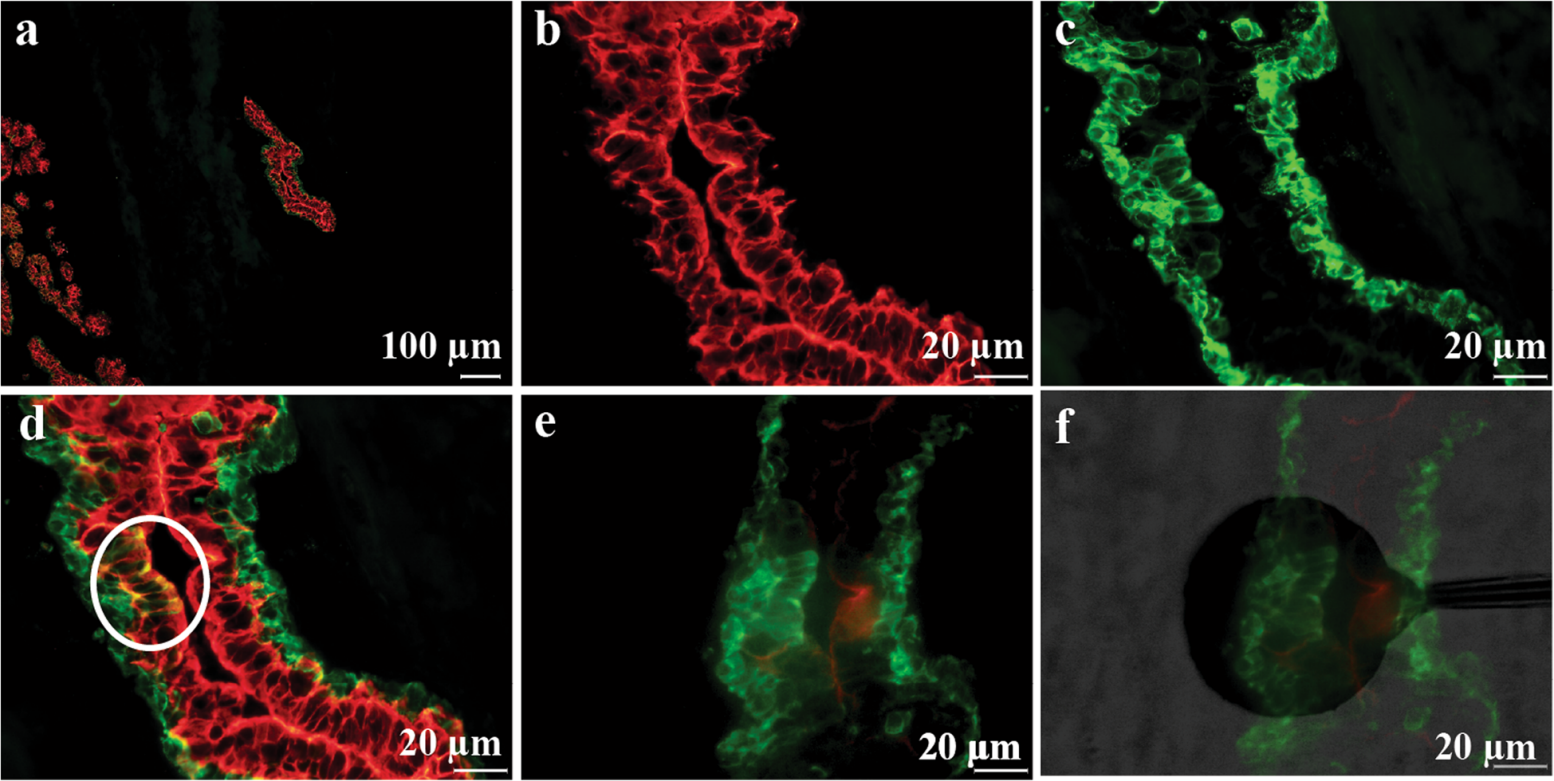
Example of target area identification and positioning of shadow stick during selection. **a** Overview of the target area. **b–d** Target area in close proximity. Red and green indicates K19 and K14 immunofluorescence respectively. The K19^+^/K14^+^ target cells are *encircled* in (**d**). **e** The exact same area on a consecutive neighboring slide. Note that the slide has been inverted in order to position shadow stick above cells. **f** Positioning of shadow stick during selection

Selections of phage displayed antibody libraries were performed on normal breast tissue sections containing a low frequency of K19^+^/K14^+^ cells lining the ductal lumen (Fig. 1a–d). Designation and localisation of target areas for selection and the positioning of the shadow stick above the target area are depicted in (Fig. 1e–f). A total of eight selections with the Predator single domain antibody library [22] yielded 93 clones. These were picked and cultured in a 96-well plate. In order to reduce the panel of domain antibodies for further analysis, the individual clones in the master plate were screened by monoclonal phage antibody ELISA on short term cultured primary breast cells enriched for K19^+^/K14^+^ cells (Additional file 1: Figure S1). A number of clones showed binding to these cells. In this study, we chose to focus on the domain antibody called BC5, since it exhibited the most interesting binding pattern in IHC stainings (as shown later) and, more importantly, this antibody is currently the only antibody for which the cognate antigen has been identified.

### BC5 binds K19^+^/K14^+^ cells in ELISA

**Fig. 2:**
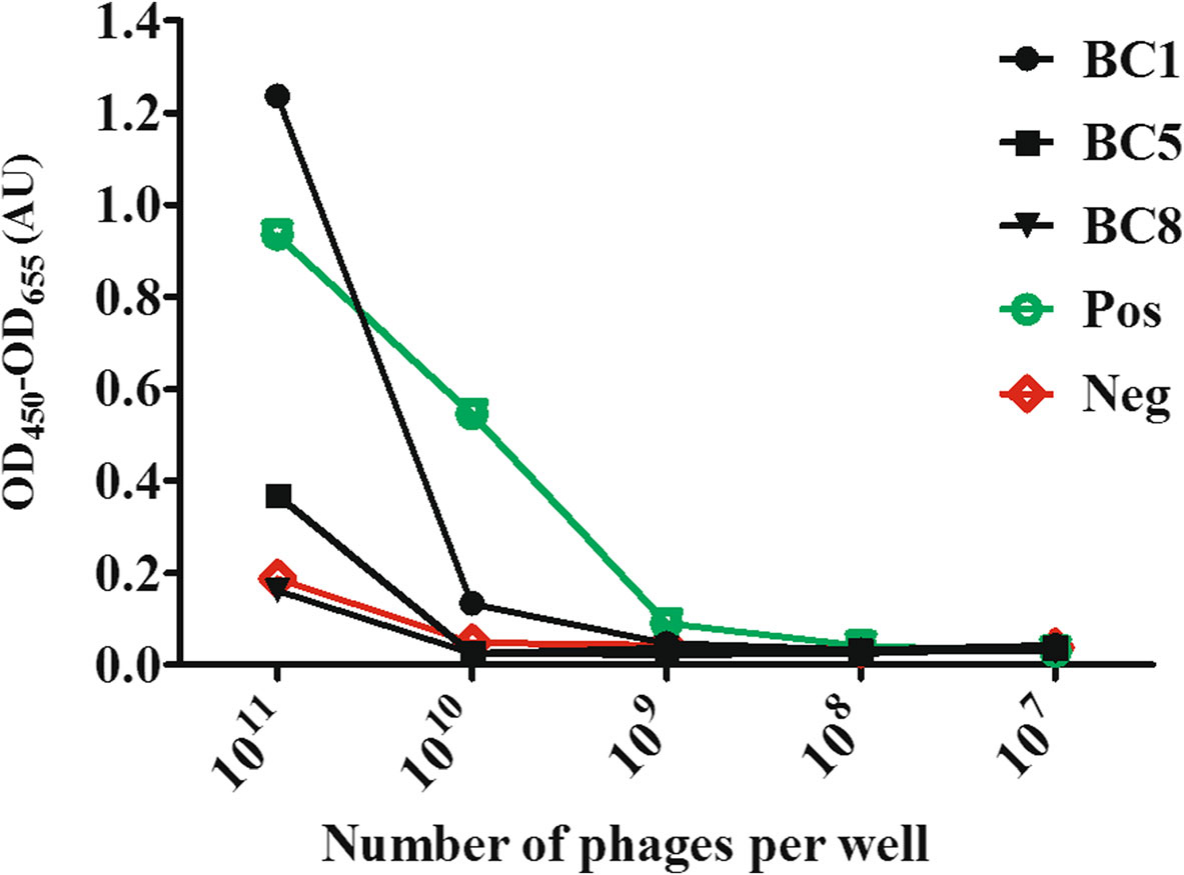
ELISA with dilution arrays of monoclonal phage antibodies on K19^+^/K14^+^ enriched cells. The y-axis depicts the ELISA signal in arbitrary units for serial dilutions of phages antibodies. The x-axis depicts the phage titer in the dilution series. Included in the figure are data for the three phage domain antibodies BC1, BC5 and BC8 as well as those of the positive and negative controls (scFvs)

To evaluate the binding of the phage domain antibody BC5, ELISA with serial dilutions of monoclonal phage antibodies was carried out on K19^+^/K14^+^ enriched cells (Fig. 2). For comparison, two other phage domain antibodies from the same selections are included in the figure. One of these is BC1, which binds strongly to K19+/K14+, but exhibits a less interesting staining pattern in IHC; it binds an antigen which is highly expressed in luminal cells and with clear staining into the stroma (Additional file 2: Figure S4). The other phage dAb, BC8, does not bind K19+/K14+ cells. The phage scFv antibody fragments 52 and DAb1 were included as positive and negative controls, respectively. BC5 binds to the K19^+^/K14^+^ enriched cells, as can be seen in Fig. 2.

### The cognate antigen of BC5 is upregulated in breast cancer

For further analysis, BC5 was sub-cloned into the vectors pET22b-MycHis and pMJ_LEXSY-rFc for expression of soluble domain antibodies with c-Myc tag and His-tag (dAb) or dimeric soluble domain antibodies with rabbit Fc-region (dAb-rFc) (data not shown). The availability of two different formats allows for detection using both the affinity tags or the Fc region of rabbit IgG, and thereby provided us with the ability to validate the binding using different formats.

**Fig. 3:**
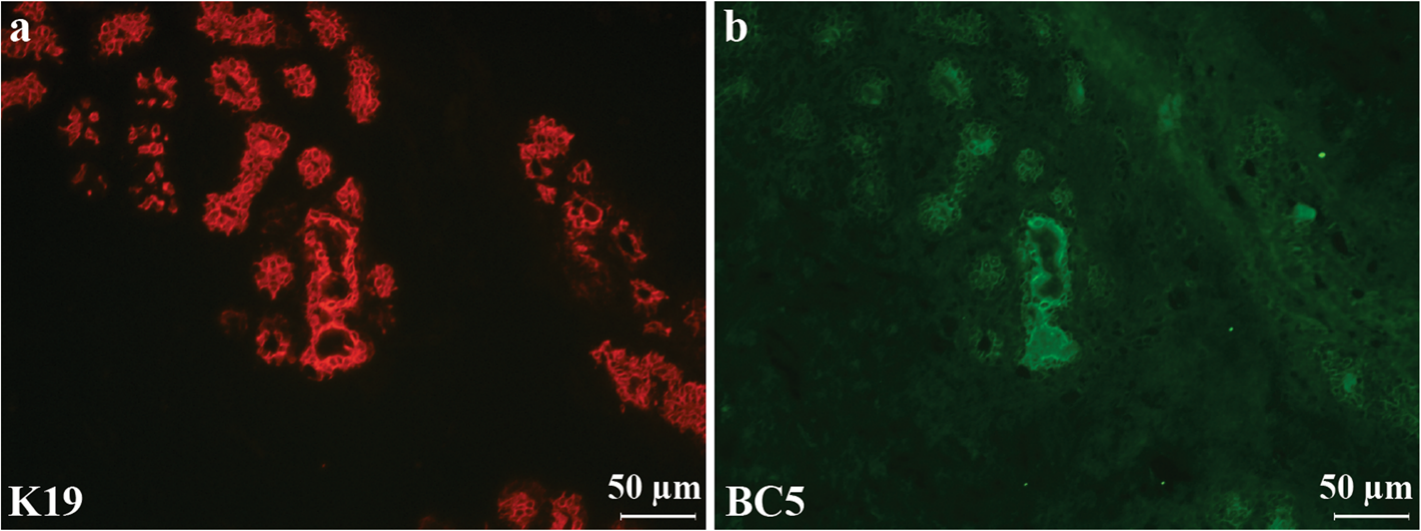
IHC with BC5 dAb-rFc and anti-K19 on normal breast tissue. **a** Detection of commercial anti-K19 (*red*). **b** Detection of BC5 (*green*) in the same area. A fraction of the K19^+^ cells was also stained by BC5. Biopsy P659

**Fig. 4:**
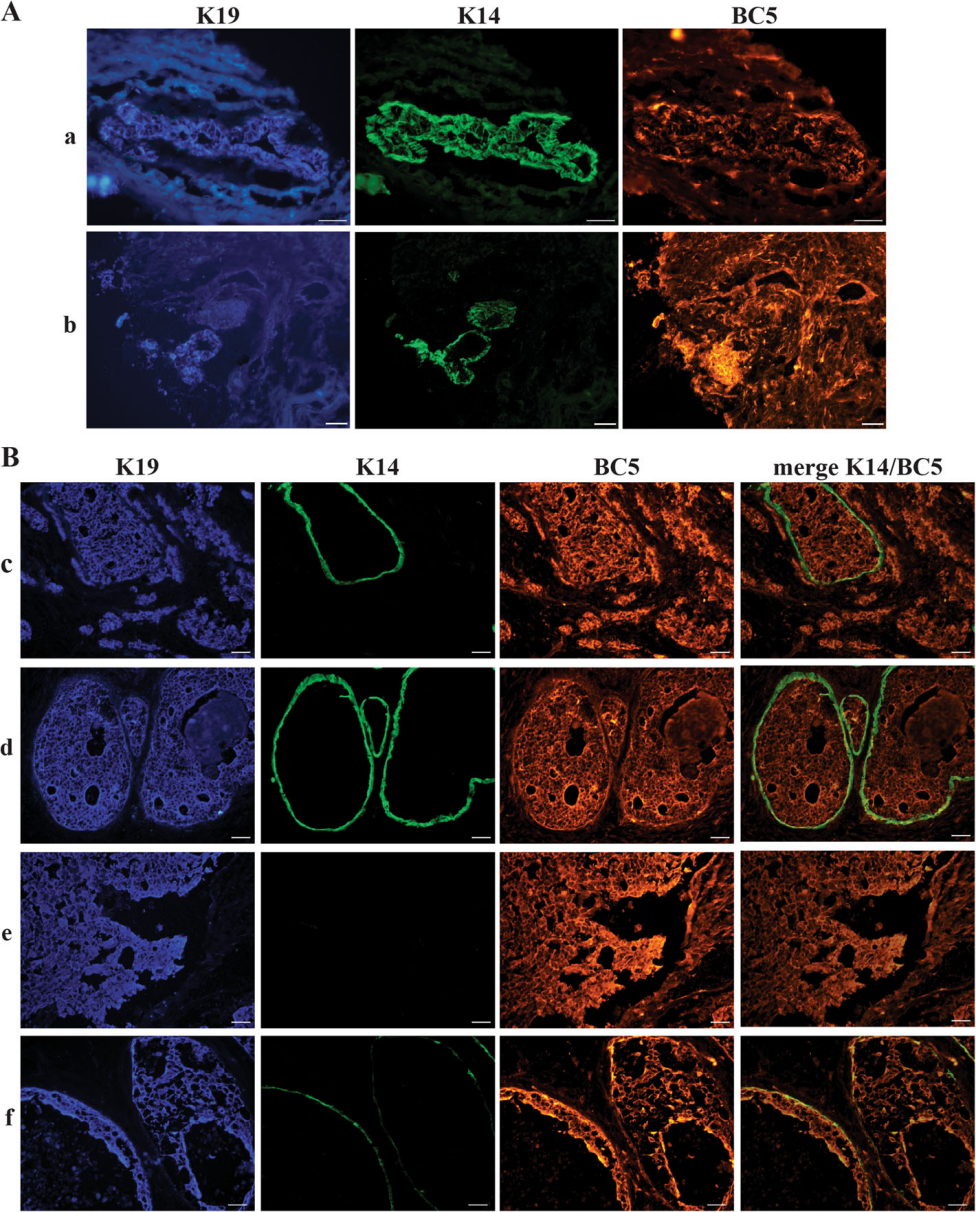
IHC on tissue microarray with BC5 dAb on normal breast and breast cancer tissue. **A** Normal tissue. The rows **a** and **b** represent two different normal breast biopsies with pictures from left to right representing staining with anti-CK19 (*blue*), anti-K14 (*green*) and BC5 dAb (*red*). **B** Breast cancer tissue. Each of the rows **c–f** represents different biopsies with pictures from left to right representing staining with anti-CK19 (*blue*), anti-K14 (*green*), BC5 dAb (*red*) and a merge of K14 and BC5. Scale bars 50 μm

IHC with both formats of soluble antibody in combination with a commercial anti-K19 antibody were carried out on five different normal breast tissue biopsies and three different breast cancer biopsies containing varying amounts of K19^+^/K14^+^ cells. The staining pattern of the domain antibody BC5 in normal tissue was compared to staining for cytokeratin 19 (Fig. 3). The staining intensities of the individual cells varied, not necessarily coinciding with the K19^+^ intensity of a given cell. In the breast cancer biopsies, a larger number of cells were stained by BC5. Furthermore, the staining followed that of K19 (not shown). Negative control stainings with secondary antibody alone or DAb1 revealed no unspecific staining (Additional file 3: Figure S2). To further assess the staining characteristics of BC5, IHC on a frozen tissue microarray with 37 different breast cancer biopsies and three normal breast biopsies was performed with the dAb format of BC5 as well as commercial mouse anti-K19 and mouse anti-K14 antibodies. Concurrent with the initial IHCs on single biopsies, the staining pattern of BC5 on normal breast tissue varied from biopsy to biopsy (Fig. 4A). The biopsy in Fig. 4A(a) shows an area with only few K19^+^ or K14^+^ cells stained by BC5, while the biopsy in Fig. 4A(b) displays an area generally stained and a cluster of intensively stained cells. However, when observing the 37 cancer biopsies, most of the cancer cells were stained by BC5 (Fig. 4B). The degree to which BC5 stained cancer cells was rather consistent from biopsy to biopsy throughout the microarray. However, before conclusive data can be obtained concerning sub-populations and relation to tumor type etc., a large scale study should be performed. Nonetheless, these results show that BC5 recognises an antigen with increased expression in some breast cancer cells, as opposed to a lower and more heterogeneous expression pattern in normal breast cells. Therefore, the cognate antigen of BC5 must be up-regulated during breast cancer tumorigenesis. In order to examine this further, the cognate antigen of BC5 was identified.

### BC5 recognizes mitochondrial ribosomal protein S18A

To exclude any cross reactivity of BC5 towards K19, an ELISA on purified K19 protein with BC5 dAb was performed. BC5 did not bind K19 (results not shown). This was further supported by the staining of K19^−^/K14^+^ cells with BC5 (Fig. 4B(d)).

**Fig. 5:**
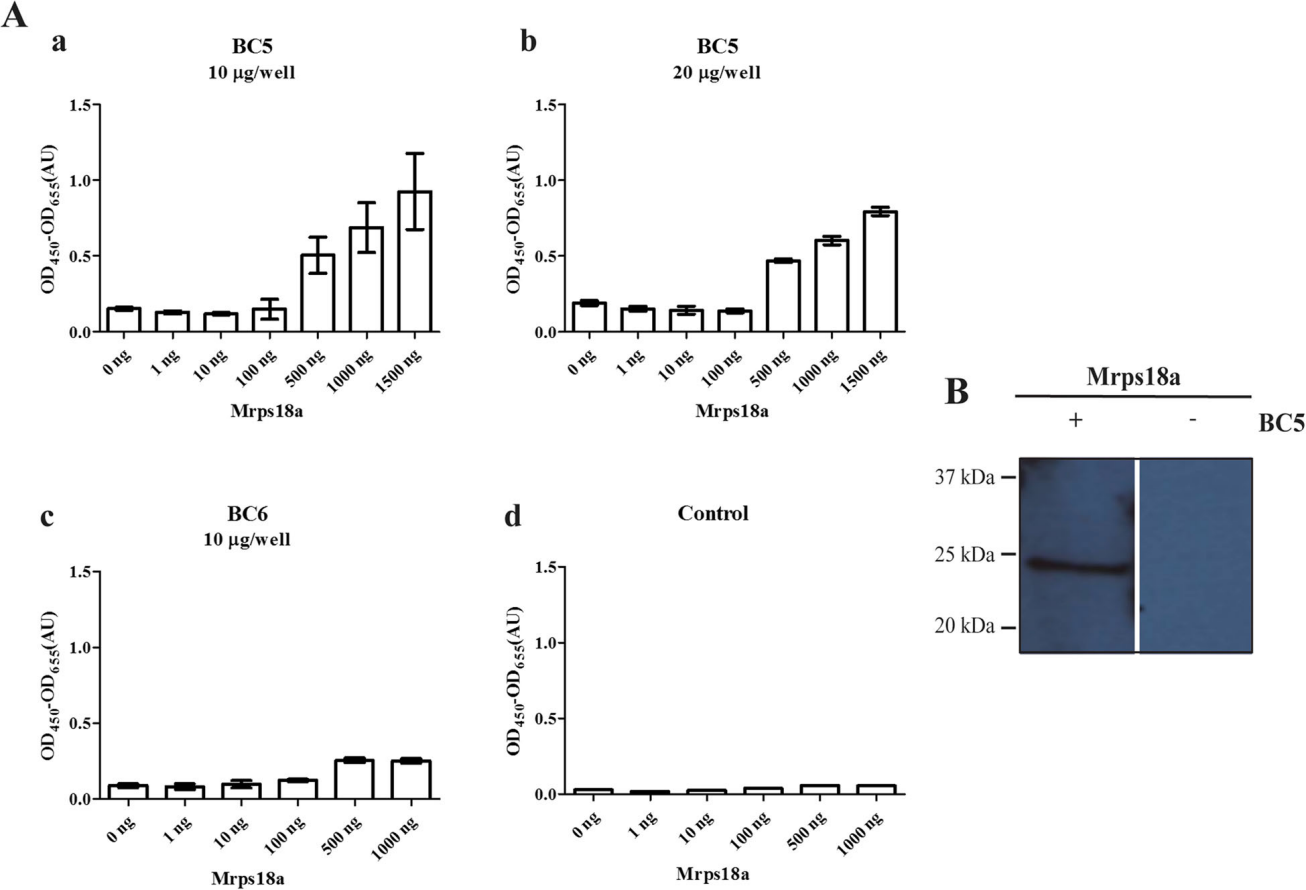
BC5 has affinity towards Mrps18a. **A** Indirect ELISA with 0–1500 ng/well coated Mrps18a. **a** Triplicates of BC5 dAb,10 μg/well. **b** Triplicates of BC5 dAb, 20 μg/well. **c** Triplicates of BC6 dAb, 10 μg/well. **d** control of unspecific background from HRP conjugated anti-c-Myc alone against coated Mrps18a. **B** Far western blot analysis of Mrps18a performed with (+) or without (−) BC5 dAb as primary antibody. Bars represent the mean ± SD. When comparing BC5 dAb, 10 μg/well with BC6 dAb, 10 μg/well the *p*-value for the signal at a Mrps18a coating concentration of 1000 ng/well is *P* < 0.01

In order to reveal the cognate antigen of BC5, a protein macroarray containing bacterially expressed cDNA clones representing more than 7000 distinct human proteins (originating from foetal brain, T-cells, lung, and colon) was incubated with the dAb format of BC5. By means of an in-house developed image analysis program, the mean intensity of the different spots on the protein macroarray was compared to the background, and a scored list ranging from the highest to lowest intensity of possible antigen hits was generated (Additional file 4: Figure S3). The macroarray was subsequently incubated with the dAb-Fc format. This was performed in order to validate the truly positive antigen hits. A single common hit was obtained, representing mitochondrial precursor ribosomal protein S18A (MRPS18A) cDNA. In order to verify the binding of BC5 to Mrps18a, the cDNA clone was sub-cloned into the pETM11 expression vector. Indirect ELISA with two different concentrations of the dAb format of BC5 on different amounts of purified Mrps18a (Fig. 5A) showed binding of BC5 to Mrps18a. BC6, another domain antibody from the current selection, was included in the experiment (Fig. 5A(d)). BC6 did not bind Mrps18a, which confirmed that the binding of BC5 to Mrps18a was related to the antigen binding region of BC5 and not to the Predator single domain scaffold. BC5 did not recognise Mrps18a in a standard western blot (results not shown). However, in a far western blot analysis of Mrps18a (Fig. 5B), BC5 showed clear recognition of Mrps18a. By performing far-western blot analysis of extracts from MCF7 cells (whole cell, cytoplasmic and mitochondrial fractions), three bands in the whole cell and two in the mitochondrial fraction are observed (Additional file 5: Figure S5). The upper band corresponds to Mrps18a, while the lower bands may constitute degradation products. The high molecular weight band observed in the cytoplasmic fraction may be a result of loading different amounts of total protein in the different lanes, as a similar band is not observed in the whole cell extract. Altogether, this indicates that BC5 recognises a conformational epitope on Mrps18a, and that the recognition is specific.

## Discussion

This study describes the identification of Mrps18a as an antigen which exhibits increased expression by some human breast cancer cells compared to normal cells. Traditionally, changes in the expression of proteins in cancer cells have been studied using both expression studies looking at the mRNA levels and proteomics using 2D gels and mass spectrometry to identify changes at the proteome level. Here, we have used the selection of recombinant phage antibody libraries as a discovery tool to identify proteins which exhibit differential expression. The putative stem cell niche on which we have selected contained K19^+^/K14^+^ cells, which are candidate cells of origin of breast cancer [14]. The principle of the selection method relies on the retrieval of phage-displayed antibody fragments binding to a minute area of interest in a tissue section. Using the shadow stick, only phages binding to the target area were able to replicate in *E. Coli*. This method yields a low output of clones from each selection compared to traditional selections, while generating a relative high frequency of phage antibodies binding to unique or upregulated antigens [23]. Important for this approach is the choice of screening material, i.e., the nature of the cells on which the selected antibodies are tested. When targeting rare cells there is always a compromise between availability of suitable cells and the extent to which they resemble the target cells. In this study, a limiting factor was the availability of primary K19^+^/K14^+^ cells for screening purposes. Here, short-term-cultured K19^+^/K14^+^ cells were used for screening. The use of short-term cultivation limited the total number of cells available for the initial ELISA testing, preventing triplicate measurements. The initial ELISA was only used as a means for defining the phage antibodies which were to be considered for further analysis. When performing ELISA with the antibody presented on the phage particle on cultured cells, care must always be taken. The data can vary greatly depending on the number of cells remaining in each 96 well after ELISA, the display level of the individual phage antibodies, the background binding due to phage coat proteins binding to the ELISA well, etc. The signal intensities in the ELISA screenings were low. However, unlike ELISA against purified proteins, low signal in ELISA against cells is expected. This is primarily because unique or upregulated antigens in the targeted cells are not necessarily expressed at high levels, or at all, in the cells used for screening. Furthermore, some of the selected phage antibodies may bind the antigens with low affinity, regardless of the relevance of the antigens in question.

From our selection, the antibody BC5 was isolated, and its cognate antigen was identified as Mrps18a. Other MRPs such as MrpL11, MrpL12 and MrpL28 have been reported to be differentially expressed in tumour cells or tissue [30, 31]. Knock-down of MRPL28 in pancreatic tumour cells resulted in decreased mitochondrial activity, increased glycolysis, and accelerated growth in vivo, a phenomenon commonly observed in cancer cells (the Warburg effect) [31]. This shows that changes in the expression of Mrps affect the mitochondrial metabolism and thereby many of the parameters that control cell state (and, in turn, cancer progression).

Furthermore, it has been observed that some Mrps have functions in addition to being involved in mitochondrial translation. For example, Mrps29, another protein component of the small mitochondrial ribosomal subunit, binds GTP and may be involved in the control of apoptosis [32, 33]. In addition, it has been reported that Mrps18b specifically binds to the viral transforming protein, EBNA-6, which targets Mrps18b to the nucleus in EBV-transformed lymphoblastoid cells. In the nucleus, Mrps18b binds retinoblastoma protein, which releases e2F1. Hence, Mrps18b may facilitate a lift of the RB-dependent block of S-phase entry [34].

A specific role for Mrps18a in cancer has not been identified until now. Due to the low level of sequence similarity in the S18 protein family, functional conservation cannot be assumed.

At present, the focus of research is turning towards elucidating the role of mitochondria in cancer. Breast cancer markers and cancer markers in general are required for the further advancement of diagnostic and treatment strategies.

## Conclusion

This study demonstrates the application of phage display as a tool for the discovery of proteins which change their expression in breast cancer. The shadow stick method allows the selection of antibodies binding to antigens expressed in specific areas within a given tissue, e.g. a stem cell niche. In this study, we identified Mrps18a as a putative breast cancer marker using this method. IHC analysis showed that Mrps18a is upregulated in human breast cancer cells compared to normal cells, indicating that Mrps18a may be involved in oncogenesis. Further investigation will clarify the role of Mrps18a in oncogenesis and its relevance as a clinical biomarker.

## Additional files

Additional file 1: Figure S1. Screening by monoclonal phage antibody ELISA on K19^+^/K14^+^ enriched breast cells. Phage antibodies showing the highest signal are presented. The y-axis depicts the ELISA signal in arbitrary units. The x-axis depicts clone name of the phage antibody. (TIF 207 kb)

Additional file 2: Figure S4. IHC with BC1 dAb and anti-K14. a, Detection of BC1 (red). b, Detection of commercial anti-K14 (green) in the same area. c, overlay of a and b. Biopsy P727. (TIF 291 kb)

Additional file 3: Figure S2. IHC controls. a–c, Example of control staining with secondary antibody Anti-C-Myc-Cy3 (red) alone, on cancer tissue (P757). From left to right the pictures show immunofluorescence in the blue, green and red range respectively. d–e, Example of control staining with the scFv antibody epsilon, which also was used as a negative control during screening and validation of selected antibodies. From left to right the pictures show immunofluorescence in the blue, green and red range respectively. (TIF 4987 kb)

Additional file 4: Figure S3. Screenshot of image analysis of protein macroarray using the in-house designed program. Upper part: A scan imaged of the protein macroarray membrane with anenlarged area to depict the pattern of proteins spotted on the membrane. Lower part: Example of the generated list of possible antigen hits. (TIF 14575 kb)

Additional file 5: Figure S5. Far-western blot of MCF7 cells using BC5 sdAb-rFc as primary antibody and swine anti-rabbit(HRP) as seconday antibody. Lane 1 contain whole extracts of MCF7 cells while Lane 2 and Lane 5 contain the cytoplasmic and mitochondrial fraction of MCF7 cells. (TIF 291 kb)

## Abbrevations

18IM: Immortalised primary rat embryonic fibroblast cell line over expressing mrps18b
HRP: Horseradish peroxidase
IHC: Immunohistochemestry
K14: Cytokeratin 14
K19: Cytokeratin 19
Mrp: Mitochondrial ribosomal proteins
Mrps18a: Mitochondrial ribosomal protein s18a
