# Supplementary figures and images for: Upregulation of Mrps18a in breast cancer identified by selecting phage antibody libraries on breast tissue sections
Source: BMC Cancer. 2017 Jan 5;17:19. doi: 10.1186/s12885-016-2987-5 (PMC5376696; doi:10.1186/s12885-016-2987-5)

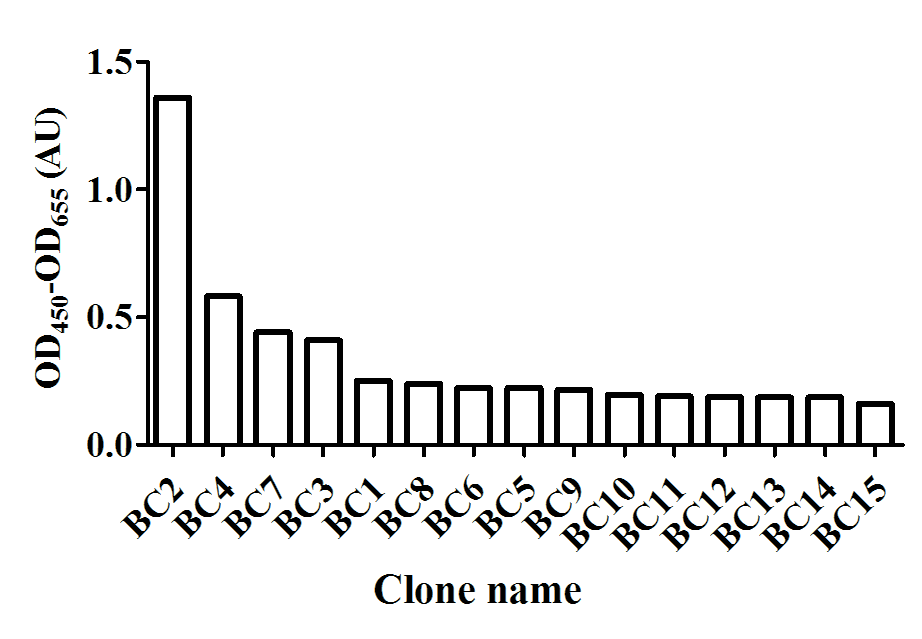

Supplement: Supplementary file 1 — Screening by monoclonal phage antibody ELISA on K19+/K14+ enriched breast cells. Phage antibodies showing the highest signal are presented. The y-axis depicts the ELISA signal in arbitrary units. The x-axis depicts clone name of the phage antibody. (TIF 207 kb) [file 12885_2016_2987_MOESM1_ESM.tif]

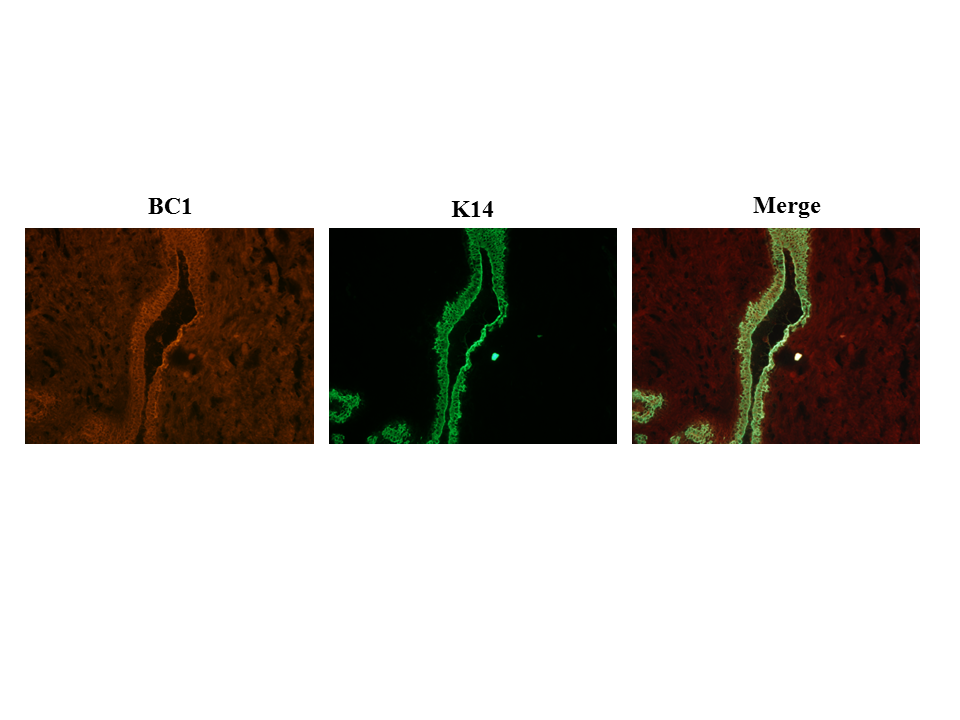

Supplement: Supplementary file 2 — IHC with BC1 dAb and anti-K14. a, Detection of BC1 (red). b, Detection of commercial anti-K14 (green) in the same area. c, overlay of a and b. Biopsy P727. (TIF 291 kb) [file 12885_2016_2987_MOESM2_ESM.tif]

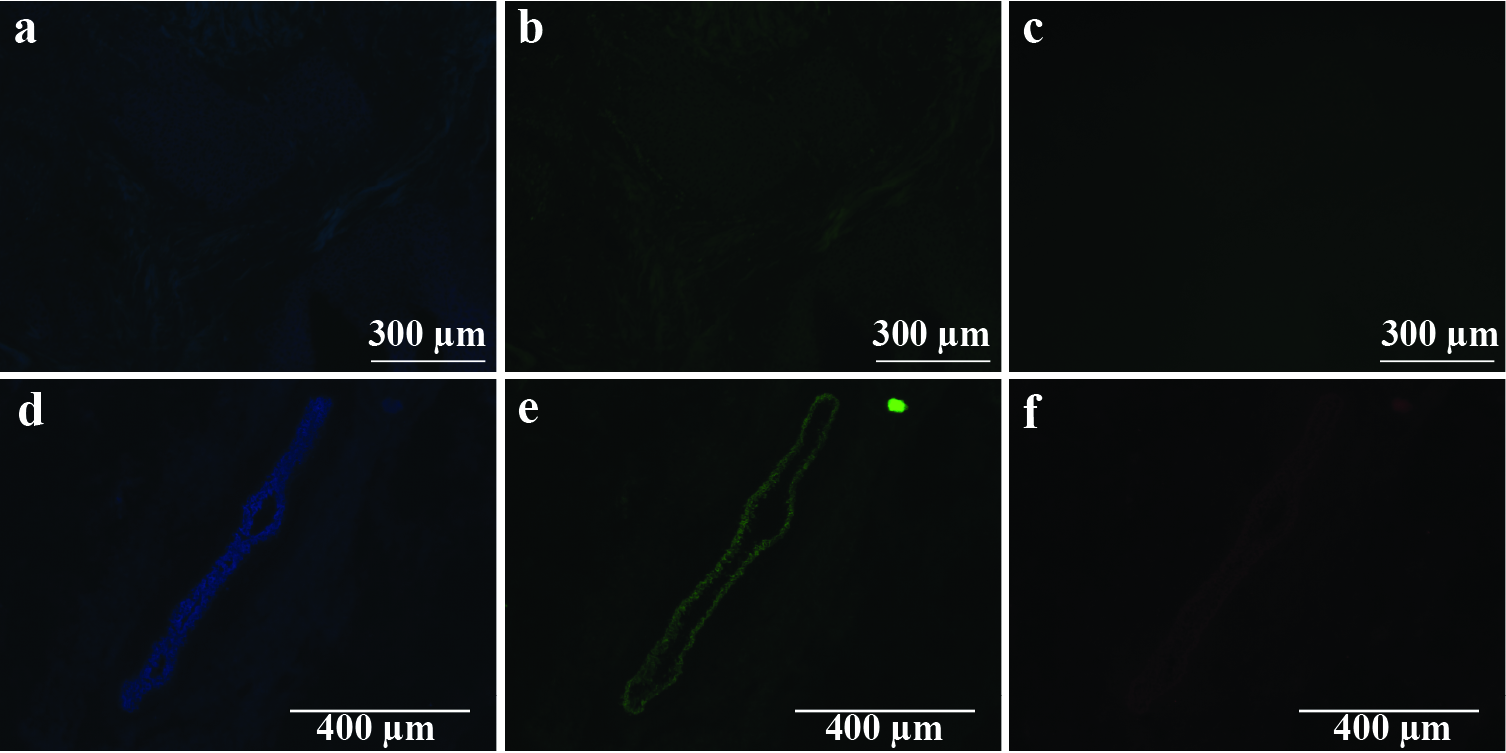

Supplement: Supplementary file 3 — IHC controls. a–c, Example of control staining with secondary antibody Anti-C-Myc-Cy3 (red) alone, on cancer tissue (P757). From left to right the pictures show immunofluorescence in the blue, green and red range respectively. d–e, Example of control staining with the scFv antibody epsilon, which also was used as a negative control during screening and validation of selected antibodies. From left to right the pictures show immunofluorescence in the blue, green and red range respectively. (TIF 4987 kb) [file 12885_2016_2987_MOESM3_ESM.tif]

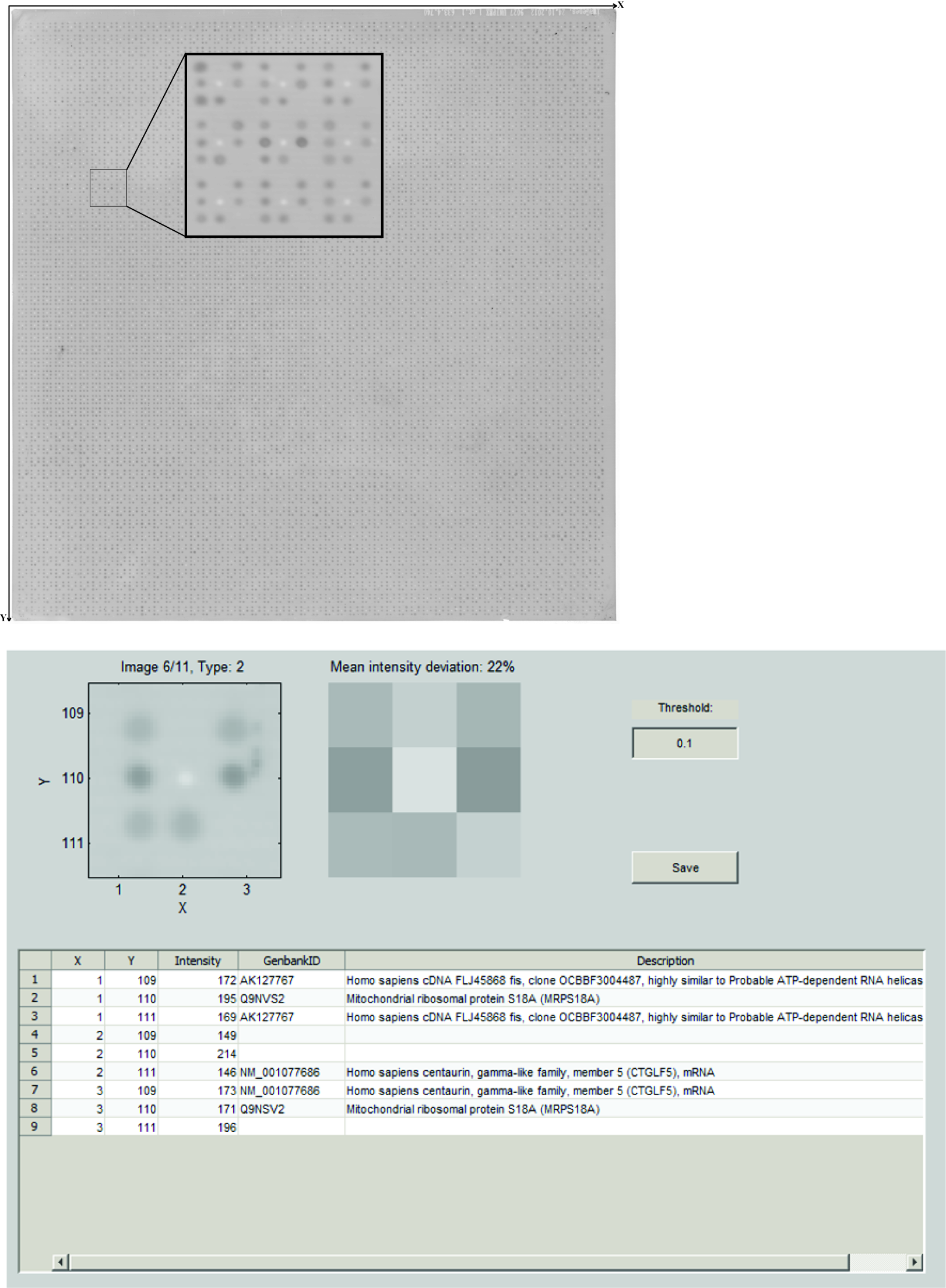

Supplement: Supplementary file 4 — Screenshot of image analysis of protein macroarray using the in-house designed program. Upper part: A scan imaged of the protein macroarray membrane with anenlarged area to depict the pattern of proteins spotted on the membrane. Lower part: Example of the generated list of possible antigen hits. (TIF 14575 kb) [file 12885_2016_2987_MOESM4_ESM.tif]

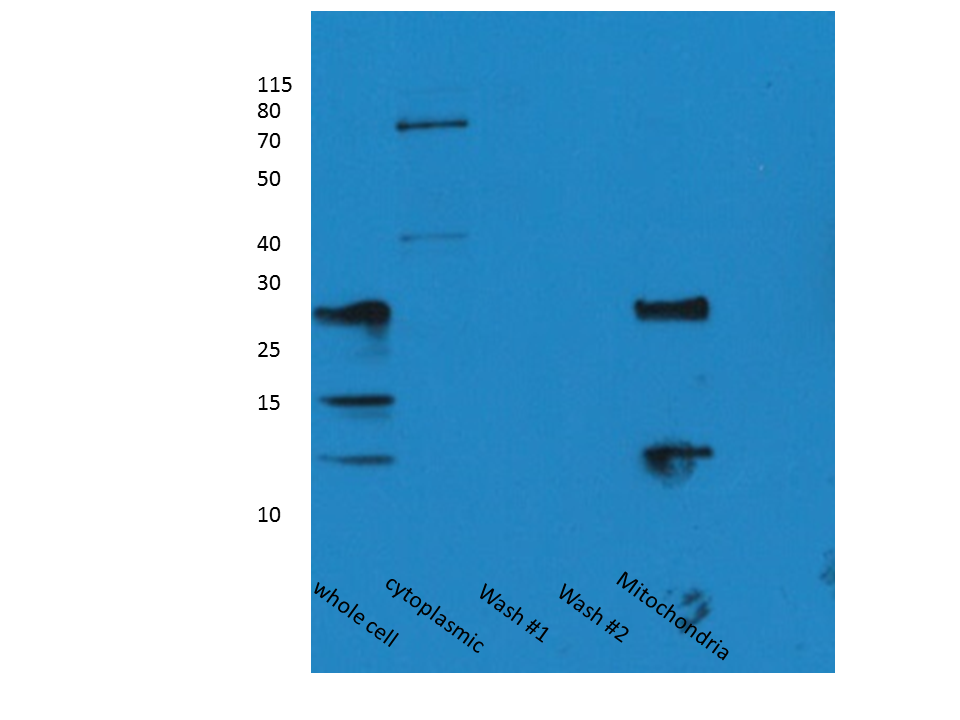

Supplement: Supplementary file 5 — Far-western blot of MCF7 cells using BC5 sdAb-rFc as primary antibody and swine anti-rabbit(HRP) as seconday antibody. Lane 1 contain whole extracts of MCF7 cells while Lane 2 and Lane 5 contain the cytoplasmic and mitochondrial fraction of MCF7 cells. (TIF 291 kb) [file 12885_2016_2987_MOESM5_ESM.tif]
